# Real-world experience with 0.2 *μ*g/day fluocinolone acetonide intravitreal implant (ILUVIEN) in the United Kingdom

**DOI:** 10.1038/eye.2017.125

**Published:** 2017-07-24

**Authors:** C Bailey, U Chakravarthy, A Lotery, G Menon, J Talks, for the Medisoft Audit Group, Clare Bailey, Aintree Kamal, Faruque Ghanchi, Calderdale Khan, Robert Johnston, Martin McKibbin, Atul Varma, Bushra Mustaq, Christopher Brand, James Talks, Nick Glover,

**Affiliations:** 1 Department of Ophthalmology, Bristol Eye Hospital, University Hospitals Bristol NHS Foundation, Bristol, UK; 2 Belfast Health and Social Care Trust, Belfast, UK; 3 University Hospital Southampton, Southampton, UK; 4 Frimley Park Hospital, Frimley, Surrey, UK; 5 Royal Victoria Hospital Newcastle upon Tyne, Newcastle Upon Tyne, UK

## Abstract

**Aims:**

To compare safety outcomes and visual function data acquired in the real-world setting with FAME study results in eyes treated with 0.2 *μ*g/day fluocinolone acetonide (FAc).

**Methods:**

Fourteen UK clinical sites contributed to pseudoanonymised data collected using the same electronic medical record system. Data pertaining to eyes treated with FAc implant for diabetic macular oedema (DMO) was extracted. Intraocular pressure (IOP)-related adverse events were defined as use of IOP-lowering medication, any rise in IOP>30 mm Hg, or glaucoma surgery. Other measured outcomes included visual acuity, central subfield thickness (CSFT) changes and use of concomitant medications.

**Results:**

In total, 345 eyes had a mean follow-up of 428 days. Overall, 13.9% of patients required IOP-lowering drops (included initiation, addition and switching of current drops), 7.2% had IOP elevation >30 mm Hg and 0.3% required glaucoma surgery. In patients with prior steroid exposure and no prior IOP-related event, there were no new IOP-related events. In patients without prior steroid use and without prior IOP-related events, 10.3% of eyes required IOP-lowering medication and 4.3% exhibited IOP >30 mm Hg at some point during follow-up. At 24 months, mean best-recorded visual acuity increased from 51.9 to 57.2 letters and 20.8% achieved ≥15-letter improvement. Mean CSFT reduced from 451.2 to 355.5 *μ*m.

**Conclusions:**

While overall IOP-related emergent events were observed in similar frequency to FAME, no adverse events were seen in the subgroup with prior steroid exposure and no prior IOP events. Efficacy findings confirm that the FAc implant is a useful treatment option for chronic DMO.

## Introduction

Diabetic macular oedema (DMO) is a serious visual complication of diabetic retinopathy, one of the leading causes of vision loss among working-age adults.^1^ Available data suggest that up to 24% of patients with diabetes develop DMO within 10 years of diagnosis.^2^

The goal of treatment is to preserve or improve vision by preventing or reducing macular swelling. Without treatment, over half of all patients who develop DMO will lose two or more lines of visual acuity within 2 years.^3^ Current first-line treatment options include focal/grid laser photocoagulation^4^ and intravitreal anti-vascular endothelial growth factor (VEGF) therapy.^5, 6, 7^

Although most patients respond to anti-VEGF therapy, a proportion of eyes do not show an improvement in vision or even lose vision. In a *post hoc* analysis of the Diabetic Retinopathy Clinical Research Network Protocol I study, 40% of eyes had an insufficient response (<5-letter improvement in best-corrected visual acuity (BCVA) from baseline) to anti-VEGF therapy at 3 months.^8^ In the Studies of Ranibizumab Injection in Subjects with Clinically Significant Macular Edema (ME) With Center Involvement Secondary to Diabetes Mellitus (RIDE and RISE), 13% of patients experienced no change or a decrease in visual acuity score at Month 24 following 0.5 mg ranibizumab therapy.^5^ Anti-VEGF therapies also require regular follow-up and usually multiple injections, resulting in a substantial treatment burden for patients and hospital eye services.^9^

Intravitreal corticosteroid implants are another effective treatment option for DMO and, as the biological activity of such implants lasts longer, a reduced frequency of injections would be expected compared with anti-VEGF monotherapy.^9, 10, 11, 12^ One long-acting steroid preparation for the management of DMO is 0.2 *μ*g/day fluocinolone acetonide (FAc) intravitreal implant (ILUVIEN, Alimera Sciences Limited, Aldershot, UK), which has proven to be efficacious for up to 36 months.^13^ However, intraocular pressure (IOP) elevations and cataract formation, which are known side effects of intravitreal corticosteroid therapy,^10, 12, 14, 15, 16, 17^ were also observed with 0.2 *μ*g/day FAc. In patients who were phakic at baseline, development of cataract was shown to occur following FAc implantation in a substantial proportion of the FAc-treated arm compared with the sham control arm, and increased IOP was also observed more frequently in the actively treated arm.^10^

In order to evaluate if the clinical trial outcomes with 0.2 *μ*g/day FAc implant were replicated in real-world settings, particularly with respect to adverse events, the present electronic medical record (EMR) based study examined safety and efficacy outcomes using data acquired from 14 clinical sites in the UK.

## Subjects and methods

All 14 clinical sites that provided data for this study used a single EMR system (Medisoft Ophthalmology, Medisoft Limited, Leeds, UK). A structured template was used as part of the retina module to record and document the findings, and thus the data that are collected are more similar to those obtained in prospective clinical trials and studies. The lead clinician and Caldicott Guardian (responsible nominee for data protection) at each NHS Hospital gave written approval for anonymised data extraction. This study was conducted in accordance with the Declaration of Helsinki and the UK’s Data Protection Act.

The present analysis was conducted using data extracted in August 2016. Data from patients who had received the 0.2 *μ*g/day FAc implant for the licenced indication of chronic DMO were identified by Medisoft Limited from the EMR system of each participating centre and extracted from each site, before being pseudoanonymised prior to amalgamation into a single dataset. Automated scripts were used to identify any patient treated with 0.2 *μ*g/day FAc implant and not flagged as a test patient. Data were then extracted from the entire record of eligible patients so that observations and treatments before and after 0.2 *μ*g/day FAc treatment were included within the analysis. A two-step approach was used to focus on DMO patients: first, non-diabetic patients were excluded and, subsequently, diabetic patients with no DMO diagnosis were excluded. Data available in the extracted set covered baseline demographics and disease characteristics including: prior treatment for DMO; the incidence of IOP elevation and management; additional ocular treatments administered post 0.2 *μ*g/day FAc implant; vision outcomes; and change in central subfield foveal thickness (CSFT).

Multiple data extracts are permitted from the EMR by the current protocol approval, up to 2018.

The range of baseline vision distribution for inclusion was not specified at the outset of the study, as this was not a clinical trial. Treatment was carried out at the clinicians’ discretion with a range of 5–85 Early Treatment Diabetic Retinopathy Study (ETDRS) letters.

IOP-related end points analysed following the 0.2 *μ*g/day FAc treatment were: percentage of treated eyes that had exhibited IOP increase ≥10 mm Hg and IOP elevation above 30 mm Hg; percentage of patients diagnosed with glaucoma or requiring IOP-lowering medication; and percentage of patients requiring trabeculoplasty and trabeculectomy/glaucoma surgery. IOP-related outcomes for patients who completed 12- and 18-month follow-up in this study were compared with the same outcomes at 12 and 18 months in the Fluocinolone Acetonide for Diabetic Macular Edema (FAME) study. Visual outcomes were also analysed from the database, including: change in vision distribution assessed by ETDRS letters; mean Best-Recorded Visual Acuity (BRVA); percentage of patients achieving ≥6/12 vision; percentage of patients with vision stability or improvement; and percentage of eyes gaining ≥15 ETDRS letters. Baseline data were included if there was a later data point for comparison. Data were excluded if only baseline data or only follow-up data were available. Baseline visual acuity (VA) is the last non-missing value taken on or before the initial date of 0.2 *μ*g/day FAc implant administration. Every non-missing VA assessment collected after the initial date of 0.2 *μ*g/day FAc administration was assigned a follow-up visit using study day number (number of days between VA assessment and initial 0.2 *μ*g/day FAc implant administration) and visit windows. In addition, drug utilisation in the 12 months prior to and following 0.2 *μ*g/day FAc implant for the treatment of DMO was analysed. End points were captured based on data captured in EMR.

Data from this real-world safety study were compared with results for patients with chronic DMO from the pivotal pre-registration FAME study at equivalent time points.^10, 11^ All comparisons were descriptive; no formal statistical analysis was undertaken.

## Results

### Study population

Data were available for 345 DMO eyes (305 patients; 40 received bilateral treatment) with a mean duration of follow-up of 428 days (range 0–919 days).

Mean age of treated patients was 68.5 years; 53.1% were male and 58.5% of patients had proliferative diabetic retinopathy at baseline. Most treated eyes (89.6%) were already pseudophakic and a further 7.2% of patients (25 eyes) received 0.2 *μ*g/day FAc implants at the same time as cataract surgery. Prior to receiving the 0.2 *μ*g/day FAc implant, 14.2% of eyes had already received IOP-lowering medication; 3.5% of eyes had IOP level >30 mm Hg at baseline and 0.3% of eyes had already required trabeculectomy or glaucoma surgery.

### Prior DMO treatment

The majority of treated eyes had received prior therapy for DMO (91.6%); 28.4% of treated eyes had received prior macular laser therapy and 84.6% of treated eyes had received at least one prior intravitreal treatment, with a mean of 7.36 prior intravitreal treatments. In patients with any prior intravitreal treatment, 32.8% had prior intravitreal steroids (29.0% received intravitreal triamcinolone; 5.5% received a dexamethasone intravitreal implant) and 78.6% had prior anti-VEGF (68.4% received ranibizumab, 21.4% bevacizumab and 1.7% aflibercept; Supplementary Tables S2 and S3).

At baseline, mean BRVA was 51.9 ETDRS letters (*n*=311). In the majority of treated eyes (84.4%) baseline vision was <70 letters.

### Incidence and management of IOP elevation

An increase in mean IOP of 3.1 mm Hg from baseline was observed during the first 12 months after 0.2 *μ*g/day FAc implantation, followed by a decrease over the subsequent 12–30 months. The mean IOP at baseline was 15.7 mm Hg, which increased to a maximum of 19.2 mm Hg at 12 months. Following this, a decrease was observed to 18.3 mm Hg at 24 months and 15.4 mm Hg at 30 months (Supplementary Figure S1).

At last observation, 13.9% of patients in the current study required IOP-lowering drops (including patients who changed existing/received additional IOP-lowering medication following 0.2 *μ*g/day FAc treatment, as well as those requiring drop initiation); 7.2% experienced an IOP elevation above 30 mm Hg and there was a single patient (0.3%) who required IOP-lowering surgery (this patient had a prior IOP-related event). The overall incidence of glaucoma (diagnosis based on report of glaucomatous cupping/notching at optic disc exam) was 1.2%.

Examining the trend over time, by 18 months the incidence of IOP increase ≥10 mm Hg was 24.4% and the rate of IOP elevation above 30 mm Hg was 13.4%. For chronic DMO patients in the FAME study, at equivalent time points, these rates were 24.9 and 10.5%, respectively. In the current study, the incidence of glaucoma at 18 months was 2.4% with 22.0% of eyes requiring emergent IOP-lowering medication while, in the FAME study, the incidence at this time point was 1.4%, with 26.3% of eyes requiring IOP-lowering treatments (Table 1; 12-month comparisons are shown in Supplementary Table S4).

### Impact of prior steroid use or prior IOP-related event

**Figure 1:**
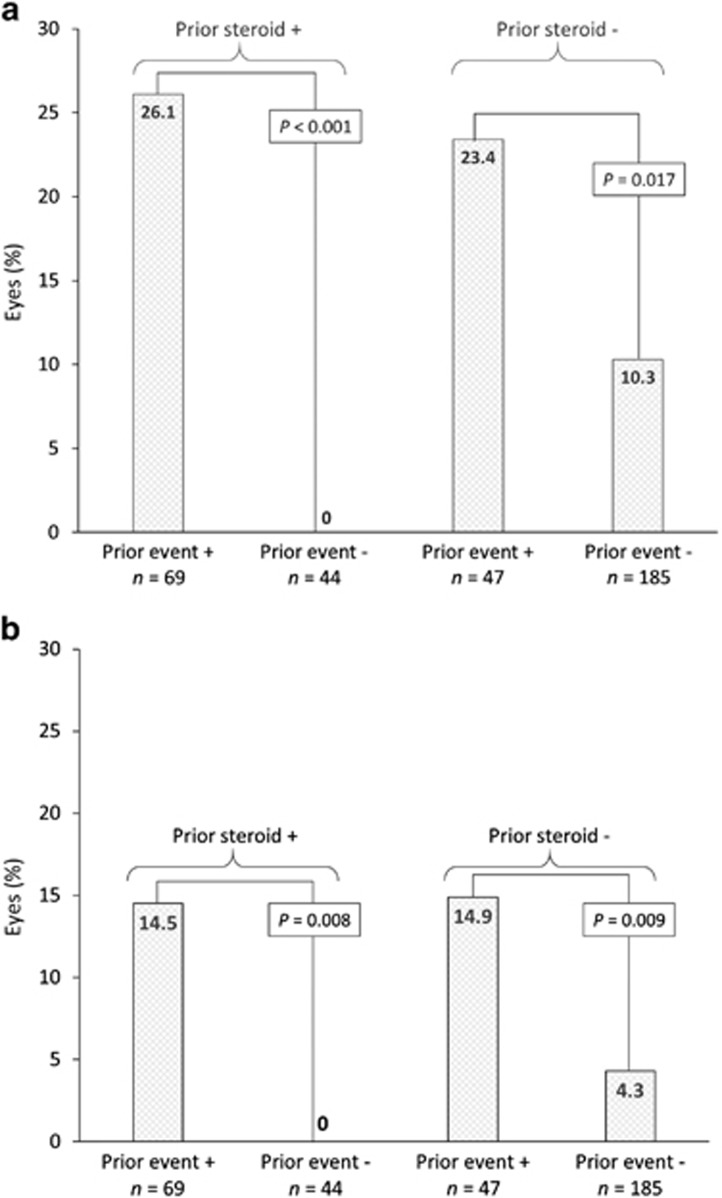
Predictors, based on prior intraocular pressure (IOP)-related event (+) or no prior IOP-related event (-) for (a) emergent IOP-lowering medications and (b) IOP peak >30 mm Hg after 0.2 *μ*g/day fluocinolone acetonide implant. Prior event is defined as report of IOP elevation, glaucoma, trabeculoplasty, glaucoma surgery, trabeculectomy, any IOP ≥21 mm Hg, IOP increase≥10 mm Hg, or report of any IOP-lowering medication.

Prior to the 0.2 *μ*g/day FAc implant, 14.2% of eyes were receiving IOP-lowering medication. Of treated eyes in the study, 33.6% had prior IOP-related events; 59.5% of these events were reported in patients without prior steroid use. In patients with prior steroid use but no prior event, 0% reported any emergent IOP-lowering medication or had an IOP elevation above 30 mm Hg. Requirement for IOP-lowering medication and IOP elevation above 30 mm Hg were both more common in eyes that had a prior IOP-related event (Figure 1). IOP-related events by history of prior IOP-related event are further described in appendix (Supplementary Table S5).

### Additional ocular treatments administered post FAc injection

The majority of eyes (64.3%) were given no additional treatments for DMO post FAc injection. Where used, additional treatments included macular laser therapy (6.4%, *n*=22), intravitreal treatment (32.2%, *n*=111) and retreatment with the FAc implant (0.53%, *n*=2). Of the eyes that received additional intravitreal treatment post FAc implant, 4 (1.2%) received bevacizumab; 47 (13.6%) aflibercept; 61 (17.7%) ranibizumab; 8 (2.3%) dexamethasone; and 8 (2.3%) triamcinolone. Overall, the mean number of intravitreal injections was 7.4 before FAc administration and 4.4 after FAc administration. For specific additional intravitreal treatments post FAc implant, the mean number of injections was 2.5 for bevacizumab; 4.0 for aflibercept; 4.5 for ranibizumab; and 1.3 for triamcinolone. One patient retreated with 0.2 *μ*g/day FAc injection had a concomitant uveitis diagnosis; the other patient had a history of pars plana vitrectomy 2 months before the second injection.

### Visual outcomes

**Figure 2:**
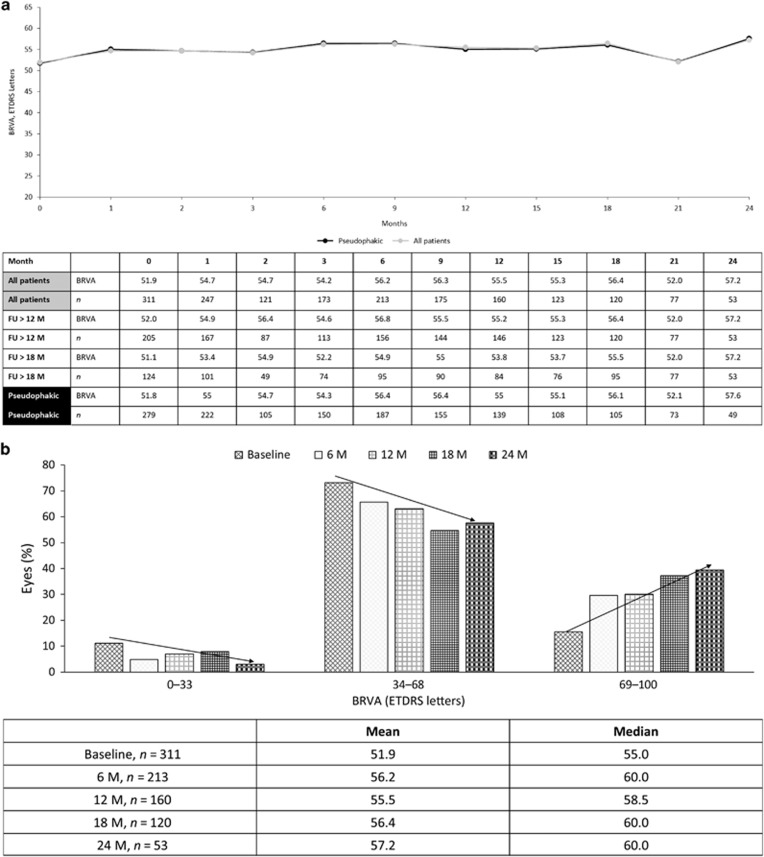
Best-recorded visual acuity (BRVA) over time^a^ in all patients and pseudophakic patients alone (a) and vision distribution over time with 0.2 *μ*g/day fluocinolone acetonide implant (b). ^a^Baseline data were included, if there was a later data point for comparison. Data were excluded, if only baseline data or only follow-up data were available. BRVA, best-recorded visual activity; ETDRS, Early Treatment Diabetic Retinopathy Study; FU, follow-up; M, month.

Mean BRVA for eyes with baseline and at least one later data point for comparison increased from 51.9 letters at baseline (*n*=311) to 56.4 letters at 18 months (*n*=120). At 24 months, mean BRVA was 57.2 letters (*n*=53). A similar trend was also demonstrated in the subgroup of pseudophakic patients. BRVA gain was maintained in patients with 12 or 18 months’ follow-up (Figure 2a). Although the majority of patients had BRVA of 34–68 letters at all time points, the distribution of VA change improved after treatment with the 0.2 *μ*g/day FAc implant, with more patients having BRVA of 69–100 letters at 18 and 24 months than prior to the implant (Figure 2b). Patients who received ≥1 intravitreal injection post FAc injection (*n*=105) had a mean BRVA of 51.8 letters at baseline. This increased by 3.2 letters to 55.0 letters at last observation. Patients who did not receive any additional treatments post FAc injection (*n*=206) had a mean BRVA of 52.0 letters at baseline. This increased by 3.0 letters to 55.0 letters at last observation.

### Vision stability, improvement and loss

The percentage of eyes with vision stability or improvement (defined by any gain or any loss less than four letters from baseline) was 78.7% at 12 months (*n*=160), 81.6% at 18 months (*n*=120) and 86.7% at 24 months (*n*=53), and remained above 74.0% over the entire period of analysis. The proportion of patients with 6/12 vision or better increased from 18.1% at baseline to 39.2% at 18 months and 39.6% at 24 months, and remained above baseline over the period of analysis (Table 2).

### Change in central subfield foveal thickness

Mean CSFT at baseline was 451.2 *μ*m (*n*=54). A mean reduction in CSFT of 95.7 *μ*m was observed over the period of analysis (*P*<0.001), with a mean CSFT at last observation of 355.5 *μ*m.

### Treatments used before and after 0.2 *μ*g/day FAc implant

Overall, 85.7% of eyes were treated with any intravitreal treatment or laser (ie, anti-VEGFs, steroids and laser) in the 12 months prior to receiving 0.2 *μ*g/day FAc implant and the overall use decreased to 32.8% in the 12 months after 0.2 *μ*g/day FAc implant. Utilisation of anti-VEGF treatment decreased by 51.3% laser treatment decreased by 2.1% and steroid treatment decreased by 2.5% in the 12 months after 0.2 *μ*g/day FAc implant, compared with the 12 months before. Table 3 compares all individual treatments recorded in the 12 months after 0.2 *μ*g/day FAc implant with those in the 12 months prior to 0.2 *μ*g/day FAc implant.

## Discussion

This study, which used the highly structured EMR data from 14 UK retina centres, was undertaken to assess real-life outcomes, with a particular focus on IOP-related adverse events, following the use of 0.2 *μ*g/day FAc implant for the treatment of chronic DMO. In this cohort, on average, the majority of treated eyes had moderate visual impairment at entry into this database and had received intravitreal therapy, including anti-VEGFs and other corticosteroids, before treatment with the FAc implant. The main finding of our analysis was the demonstration of a favourable safety profile, with important improvements in VA and retinal morphology.

The range of VA of eyes at the time of 0.2 *μ*g/day FAc implant treatment that were included in the EMR dataset was different and vastly broader than of the FAME study^10, 11^ and in other landmark clinical trials of intravitreal therapies for DMO.^5, 6, 12^ In the real-life setting, we observed that treatment was initiated in eyes with VA ranging from 5 to 85 ETDRS letters, compared with a range of 19–68 ETDRS letters in the FAME study and 24–73 ETDRS letters in the RISE/RIDE trial.

Overall, the side-effect profile demonstrated in this study was in line with the known safety profile for intravitreal corticosteroids. The majority of IOP increases did not require medical management, as demonstrated by only 13.9% of patients requiring IOP-lowering drops (including patients who changed existing/received additional IOP-lowering medication since 0.2 *μ*g/day FAc treatment, as well as those requiring drop initiation) and 0.3% requiring glaucoma surgery at last observation. In the overall study population, the increase in mean IOP observed was gradual and remained under 20 mm Hg throughout the period of analysis. Our real-world data show that the proportion of patients requiring IOP-lowering medication or glaucoma surgery at 18 months was lower than that in the FAME study at 18 months. In addition, the incidence of glaucoma was low in this study (2.4% in 18-month completers). Future analyses of data collected following 0.2 *μ*g/day FAc treatment could include exploration of the relationship between IOP and lens status and thus provide further insight into the extent of the effect of cataract removal on IOP.

Our data also show that 0.2 *μ*g/day FAc implant has a favourable safety profile for patients without prior IOP-related events. Patients without an IOP elevation in response to a steroid challenge may be particularly good candidates for this technology. IOP rise is frequent in DMO patients and may not always be related to steroid use, as demonstrated by the fact that 59.5% of the patients that had reported prior IOP-related events did not have a history of prior steroid use. Emergent IOP-lowering medication or IOP elevation above 30 mm Hg following treatment with 0.2 *μ*g/day FAc was more frequently reported in patients with prior IOP-related events. Based on the data from this study, for patients who had not previously received steroid and were without prior IOP-related events, only 4.3% developed IOP elevation above 30 mm Hg and 10.3% required IOP-lowering medication following FAc treatment, indicating a low likelihood of IOP complications in this patient population.

Although data on macular thickness were not comprehensively recorded, a reduction in mean CSFT was observed following treatment with the FAc implant. Overall, these results are in line with those of recent case reports and other studies of 0.2 *μ*g/day FAc implant for the treatment of DMO in real-world settings,^18, 19, 20^ and highlight the potential additional value that can be gained by switching to the FAc implant in patients with a suboptimal response to other treatment options.

The strengths of a database study such as this are the diversity of the patient population, in terms of baseline clinical characteristics, and the fact that patients were treated with 0.2 *μ*g/day FAc implant in real-world settings; thus enabling a more direct translation of the study findings to routine clinical practice. For example, in contrast to clinical trials, patients with ocular comorbidities such as epiretinal membrane and vitreomacular traction were not excluded and no restrictions were placed on systemic parameters, such as HbA1c and blood pressure; despite this, comparable efficacy to clinical trials was observed.

A potential limitation is the interim and open-label nature of this analysis. Although the data were entered prospectively, they were analysed retrospectively and no further information could be obtained if there were missing data points, such as optical coherence tomography thickness; although subsequent analyses are likely to provide further information on the long-term tolerability and efficacy of 0.2 *μ*g/day FAc in real-world settings. Additionally, validation of disease state was not carried out and quality of data was dependent on completeness and accuracy of electronic records. A confounding factor to consider when using real-world data is that VA is often measured with the patients’ habitual correction (if any) rather than objective refractions at each visit. This is likely to underestimate the actual changes in vision. However, it may better reflect the visual outcomes that patients actually experience following treatment and is reassuring for the outcomes that ophthalmologists can advise their patients to expect.

Treatment with a single 0.2 *μ*g/day FAc implant has the potential to considerably improve patients’ quality of life, owing to improved vision and a decreased requirement for additional therapies/visits. Despite poor starting vision and receiving prior therapy, treatment with the FAc implant was associated with sustained or improved vision for 86.7% of patients at 24 months. The majority of patients did not receive additional treatments, however, it should be noted that 35.7% of patients received additional treatments post FAc injection. Nevertheless, visual outcomes were similar for patients regardless of if they received additional intravitreal treatment or not. As most patients had received intravitreal anti-VEGF prior to the FAc implant, the visual improvements on top of previous improvements in VA are important. These results are especially noteworthy, since the patient population in this study may have been at a later stage of disease than was studied in the RISE and RIDE studies for intravitreal ranibizumab.^5, 6, 21^ Another important quality of life consideration is maintenance of functional vision; the legal minimum requirement for driving in the UK is binocular VA of 6/12.^22^ The proportion of patients with 6/12 vision or better in the treated eye increased from 18.1% at baseline to 39.6% at 24 months, and remained above baseline over the period of analysis.

In conclusion, the results of this EMR audit provide further evidence of the tolerability and efficacy of 0.2 *μ*g/day FAc for the treatment of chronic DMO in routine clinical practice. The FAc implant appears to be a valuable therapeutic approach for patients with chronic DMO refractory to other treatment options, with a predictable and manageable side-effect profile that is more favourable in the absence of prior IOP-related events.


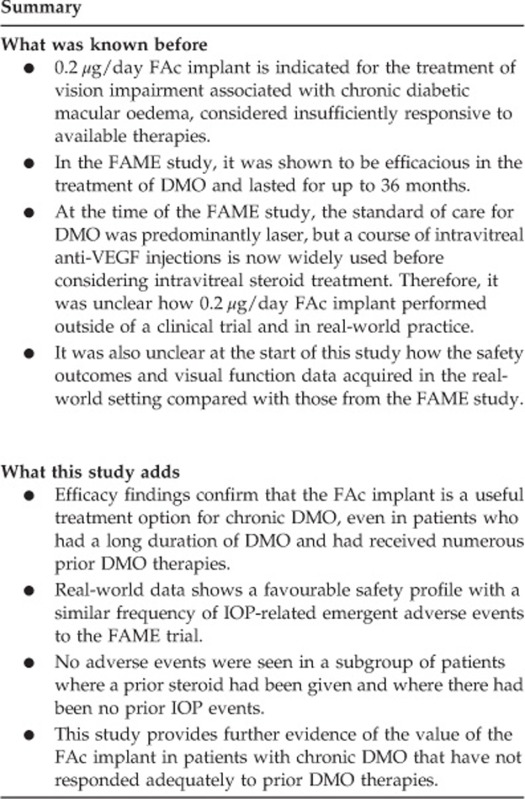


## Figures and Tables

**Table 1:** (**a**) IOP elevation and management. (**b**) Comparison of IOP elevation and management for patients completing 18 months of this study compared with 18 months in FAME study

(**a**)			
*Medisoft data*	*DMO eyes %* (n)*Mean follow-up 428 days* *n*=*345*	*Prior steroid use and no prior IOP-related event* % (n) *n*=*44*	*No prior steroid use and no prior IOP-related events* % (n) *n=185*
IOP increase of ≥10 mm Hg	15.4 (53/345)	6.8 (3/44)	9.7 (18/185)
IOP elevation above 30 mm Hg	7.2 (25/345)	0 (0/44)	4.3 (8/185)
Trabeculoplasty	0 (0/345)	0 (0/44)	0 (0/185)
Trabeculectomy/glaucoma surgery	0.3 (1/345)	0 (0/44)	0 (0/185)
Reported 'glaucoma'^a^	1.2 (4/345)	0 (0/44)	0.5 (1/185)
Emergent IOP-lowering medication^b^	13.9 (48/345)	0 (0/44)	10.3 (19/185)

(**b**)			
	*Medisoft*^c^ *18-month DMO completers* % (n) *n*=*127*	*FAME*^d^ *(cDMO patients, 18 months)* % (n) *n*=*209*	
IOP increase of ≥10 mm Hg	24.4 (31/127)	24.9 (52/209)	
IOP elevation above >30 mm Hg	13.4 (17/127)	10.5 (22/209)	
Trabeculoplasty	0 (0/127)	0 (0/209)	
Trabeculectomy/glaucoma surgery	0.8 (1/127)	1.5 (3/209)	
Reported 'glaucoma'	2.4 (3/127)	1.4 (3/209)	
Emergent IOP-lowering medication^b^	22.0 (28/127)	26.3 (55/209)	

Abbreviations: cDMO, chronic diabetic macular oedema; DMO, diabetic macular oedema; FAc, fluocinolone acetonide; IOP, intraocular pressure.

a Includes diagnosis post FAc implant reassessed by the principal investigator.

b Includes IOP-lowering medications initiated after FAc injection and addition and/or switch of medication in patients with baseline IOP-lowering medication.

c Preliminary safety findings for patients with at least 18 months of follow-up.

d Preliminary safety findings at 18 months for the FAME study.

**Table 2:** Vision changes at 12 and 24 months—vision loss, stability and improvement at 12 months and 24 months and percentage of eyes achieving [6/12 or better] following 0.2 *μ*g/day FAc implant

	*12 months (%)* *n*=*160*	*18 months (%)* *n*=*120*	*24 months (%)* *n*=*53*
Vision stability/improvement	78.7	81.6	86.7
≥15-letters gain	15.0	15.0	20.8
≥10-letters gain	28.1	32.5	34.0
≥5-letters gain	48.1	47.5	52.9
6/12 vision or better	30.0	39.2	39.6
BRVA loss of>0 letters	27.6	26.7	22.7
BRVA loss of>15 letters	6.9	5.0	5.7

Abbreviations: BRVA, best-recorded visual acuity; FAc, fluocinolone acetonide.

**Table 3:** Therapy utilisation in patients with both 12 months of follow-up before 0.2 *μ*g/day FAc implant and 12 months of follow-up after 0.2 *μ*g/day FAc implant

*Treatment*	*Use 12 months prior to FAc implant* (%)	*Use 12 months post FAc implant* (%)
Ranibizumab	66.1	18.0
Aflibercept	1.1	6.9
Bevacizumab	9.0	0
Dexamethasone implant	2.1	1.6
Panretinal photocoagulation (PRP)	5.8	6.3
Macular laser	7.4	4.8
IVTA	4.2	2.2

Abbreviations: FAc, fluocinolone acetonide; IVTA, intravitreal triamcinolone.
