# Supplementary figures and images for: Real-world experience with 0.2 μg/day fluocinolone acetonide intravitreal implant (ILUVIEN) in the United Kingdom
Source: Eye (Lond). 2017 Jul 24;31(12):1707–15. doi: 10.1038/eye.2017.125 (PMC5733285; doi:10.1038/eye.2017.125)

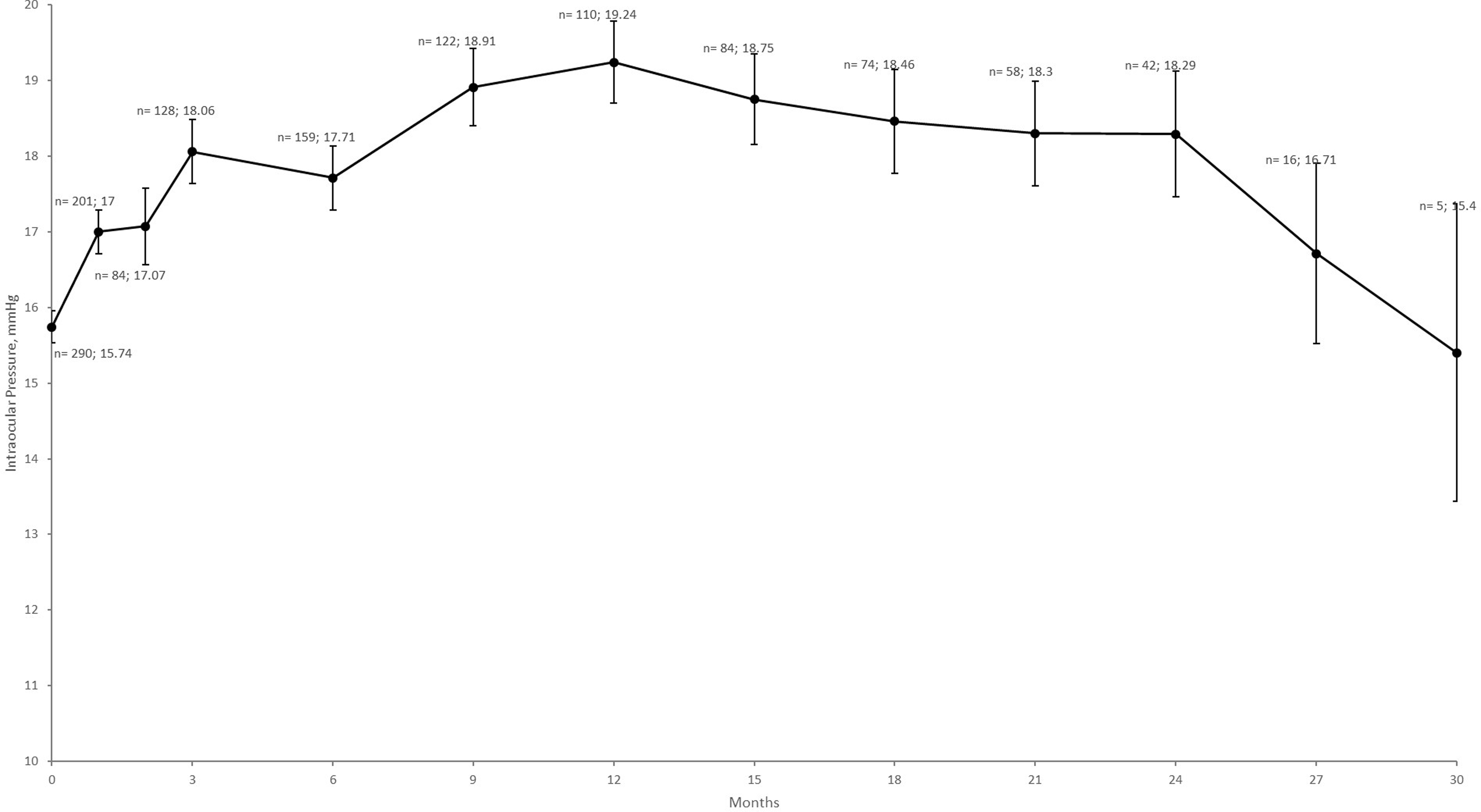

Supplement: Supplementary Figure S1 [file eye2017125x1.tif]
