# Supplementary material for: Real-world experience with 0.2 μg/day fluocinolone acetonide intravitreal implant (ILUVIEN) in the United Kingdom
Source: Eye (Lond). 2017 Jul 24;31(12):1707–15. doi: 10.1038/eye.2017.125 (PMC5733285; doi:10.1038/eye.2017.125)
Supplement: Supplementary Table S1 [file eye2017125x2.docx]

**Table S1** Medisoft Audit Group Contributors

| **Name** | **Affiliation** |
| --- | --- |
| **Dr Clare Bailey**  **(Principal Investigator)** | Bristol Eye Hospital |
| Dr Ahmed Kamal | Aintree University Hospitals |
| Prof Usha Chakravarthy | Belfast Health and Social Care Trust |
| Dr Faruque Ghanchi | Bradford Teaching Hospitals |
| Dr Rehna Khan | Calderdale Royal Hospital |
| Prof Geeta Menon | Frimley Park Hospital |
| Dr Robert Johnston | Gloucestershire Hospitals |
| Dr Martin McKibbin | Leeds Teaching Hospitals |
| Dr Atul Varma | Mid Yorkshire Hospitals |
| Dr Bushra Mustaq | BMEC |
| Dr Christopher Brand | Sheffield Teaching Hospitals |
| Dr James Talks | Newcastle Upon Tyne Hospitals |
| Prof Andrew Lotery | University Hospital Southampton |
| Dr Nick Glover | Queen Elizabeth Birmingham |
