# Supplementary material for: Real-world experience with 0.2 μg/day fluocinolone acetonide intravitreal implant (ILUVIEN) in the United Kingdom
Source: Eye (Lond). 2017 Jul 24;31(12):1707–15. doi: 10.1038/eye.2017.125 (PMC5733285; doi:10.1038/eye.2017.125)
Supplement: Supplementary Table S2 [file eye2017125x3.docx]

**Table S2** Patient demographics and baseline characteristics

| **Characteristic** | **Patients (n=305; 345 DMO eyes)** |
| --- | --- |
| Gender | 53.1% male/46.9% female |
| Mean age | 68.5 years |
| Age at time of diabetes diagnosis | 48.3 years |
| Type of diabetes, % |  |
| Type I | 16.7% |
| Type II | 76.7% |
| Not specified/missing/others | 6.6% |
| Patients under insulin therapy, % | 54.1% |
| Lens status | 89.6% pseudophakic/10.4% phakic |
| Combined cataract surgery/0.2 *µ*g/day FAc implant | 7.2% (25 eyes) |
| Mean duration of follow-up | 428 days (range 0–919) |
| Any prior macular laser or intravitreal treatment | 91.6% (316 eyes) |
| Any prior macular laser treatment | 28.4% (98 eyes) |
| Any prior intravitreal treatment | 84.6% (292 eyes), mean 7.36 treatments |
| Intravitreal steroid | 32.8% (113 eyes) |
| Intravitreal anti-VEGF | 78.6% (271 eyes) |
| Intravitreal treatments |  |
| Ranibizumab | 68.4% (236 eyes) |
| IVTA | 29.0% (100 eyes) |
| Bevacizumab | 21.4% (74 eyes) |
| Dexamethasone implant | 5.5% (19 eyes) |
| Aflibercept | 1.7% (6 eyes) |
