# Supplementary material for: Real-world experience with 0.2 μg/day fluocinolone acetonide intravitreal implant (ILUVIEN) in the United Kingdom
Source: Eye (Lond). 2017 Jul 24;31(12):1707–15. doi: 10.1038/eye.2017.125 (PMC5733285; doi:10.1038/eye.2017.125)
Supplement: Supplementary Table S3 [file eye2017125x4.docx]

**Table S3** Summary of number of patients and eyes listed by site (population: all extracted diabetic macular oedema patients receiving fluocinolone acetonide implant)

| *Site identifier* | *Patients treated,* n *(%)* | *Eyes treated,* n | *Treatments administered,* n |
| --- | --- | --- | --- |
| A | 8 (2.6%) | 8 | 8 |
| B | 56 (18.4%) | 70 | 70 |
| C | 5 (1.6%) | 6 | 6 |
| D | 4 (1.3%) | 5 | 5 |
| E | 11 (3.6%) | 11 | 12 |
| F | 30 (9.8%) | 32 | 32 |
| G | 11 (3.6%) | 11 | 11 |
| H | 27 (8.9%) | 28 | 28 |
| I | 21 (6.9%) | 26 | 26 |
| J | 26 (8.5%) | 30 | 30 |
| K | 20 (6.6%) | 20 | 20 |
| L | 46 (15.1%) | 53 | 53 |
| M | 9 (3.0%) | 10 | 10 |
| N | 31 (10.2%) | 35 | 35 |
| **TOTAL** | **305 (100.0%)** | **345** | **346** |
