# Supplementary material for: Real-world experience with 0.2 μg/day fluocinolone acetonide intravitreal implant (ILUVIEN) in the United Kingdom
Source: Eye (Lond). 2017 Jul 24;31(12):1707–15. doi: 10.1038/eye.2017.125 (PMC5733285; doi:10.1038/eye.2017.125)
Supplement: Supplementary Table S4 [file eye2017125x5.docx]

**Table S4** Preliminary safety findings at 12 months^a,b^ and 18 months^c,d^ – intraocular pressure (IOP; diabetic macular oedema [DMO] eyes only)

| *IOP related event* | *Medisoft^a^ 12-month DMO completers* N *= 210* | *FAME^b^ (cDMO patients, 12 months)* N *= 209* | *Medisoft^c^ 18-month DMO completers* N *= 127* | *FAME^d^ (cDMO patients, 18 months)* N *= 209* | |
| --- | --- | --- | --- | --- | --- |
| IOP increase of ≥ 10mmHg | 20.0% (42/210) | 20.6% (43/209) | 24.4% (31/127) | 24.9% (52/209) | |
| IOP elevation above > 30 mmHg | 11.0% (23/210) | 7.7% (16/209) | 13.4% (17/127) | 10.5% (22/209) | |
| Trabeculoplasty | 0.0% (0/210) | 0.0% (0/209) | 0.0% (0/127) | 0.0% (0/209) | |
| Trabeculectomy/glaucoma surgery | 0.5% (1/210) | 0.5% (1/209) | 0.8% (1/127) | 1.5% (3/209) | |
| Reported ‘glaucoma’ | 0.0% (0/210) | 0.5% (1/209) | 2.4% (3/127) | 1.4% (3/209) | |
| Emergent IOP-lowering medication^e^ | 18.1% (38/210) | 21.1% (44/209) | 22.0% (28/127) | 26.3% (55/209) | |
| ^a^Preliminary safety findings at 12 months for patients who complete the 12-month visit. ^b^Preliminary safety findings at 12 months for the FAME study. ^c^Preliminary safety findings at 18 months for patients who completed the 18-month visit. ^d^Preliminary safety findings at 18 months for the FAME study. ^e^Includes IOP-lowering medications initiated after fluocinolone acetonide injection and addition and/or switch of medication in patients with baseline IOP-lowering medication. | | | | |  |
