# Supplementary material for: Real-world experience with 0.2 μg/day fluocinolone acetonide intravitreal implant (ILUVIEN) in the United Kingdom
Source: Eye (Lond). 2017 Jul 24;31(12):1707–15. doi: 10.1038/eye.2017.125 (PMC5733285; doi:10.1038/eye.2017.125)
Supplement: Supplementary Table S5 [file eye2017125x6.docx]

**Table S5** Intraocular pressure (IOP)-related events after administration of fluocinolone acetonide (FAc) implant by history of IOP-related event prior to FAc implant (population: all extracted diabetic macular oedema patients receiving FAc implant with history of prior ocular steroids)

|  | *History of IOP-related events* | |  |
| --- | --- | --- | --- |
| *IOP-related events Post-FAc implant* | *No* n *(eyes) = 44* | *Yes* n *(eyes) = 69* | P*-value* |
| Glaucoma surgery | 0 (0.0%) | 1 (1.4%) | 0.422 |
| Trabeculectomy^a^ | 0 (0.0%) | 0 (0.0%) |  |
| IOP increase of 10 mmHg or more^b^ | 3 (6.8%) | 19 (27.5%) | 0.007 |
| IOP elevation to over 21 mmHg | 5 (11.4%) | 33 (47.8%) | <0.001 |
| IOP elevation to over 25 mmHg | 3 (6.8%) | 22 (31.9%) | 0.002 |
| IOP elevation to over 30 mmHg | 0 (0.0%) | 10 (14.5%) | 0.008 |
| Any treatment-emergent IOP-lowering medication^c^ | 0 (0.0%) | 18 (26.1%) | <0.001 |
| ^a^Reported as a co-pathology. ^b^Change relative to the last recorded IOP assessment prior to FAc implant. ^c^Includes IOP-lowering medications initiated post-FAc implant. *P*-values based on a Pearson chi-square test. | | | |
